# Integrating senotherapeutics into transplantation: liver reconditioning in an aging donor pool

**DOI:** 10.3389/ti.2026.16347

**Published:** 2026-06-08

**Authors:** Madita L. Buch, Hannah Esser, Himath Perera, Runshi Zheng, Sofia Ferreira-Gonzalez, Stuart J. Forbes

**Affiliations:** 1 Centre for Regenerative Medicine, Institute for Regeneration and Repair, The University of Edinburgh, Edinburgh, United Kingdom; 2 Department of Surgery, Division of Hepato-Pancreato-Biliary and Transplant Surgery, Erasmus MC Transplant Institute, University Medical Center Rotterdam, Rotterdam, Netherlands; 3 Centre for Inflammation Research, Institute for Regeneration and Repair, The University of Edinburgh, Edinburgh, United Kingdom

**Keywords:** aging, liver transplantation, machine perfusion, organ reconditioning, organ rejuvenation, senolytics, senescence

## Abstract

Our aging population is reshaping transplantation medicine. As demand for liver transplantation continues to rise, an aging donor pool presents unique challenges, with marginal organs becoming increasingly prevalent and representing a critical yet underexploited opportunity. Current selection criteria, such as chronological age, may not fully capture organ quality. A multidimensional approach that better reflects true biological aging is now more crucial than ever. Increasing evidence indicates that senescence, a hallmark of aging, influences multiple stages of transplantation, including organ procurement and preservation. Assessing senescence could provide an objective metric for evaluating organ quality. Importantly, senescence quantification could both define organ quality and guide interventions aimed at mitigating this phenomenon. This review explores the contribution of senescence to the transplant process and evaluates emerging opportunities for senescence-based assessment and therapeutic intervention. We also highlight the potential to integrate these strategies with *ex vivo* machine perfusion to quantify senescence burden, deliver targeted interventions, and functionally recondition marginal grafts, thereby expanding the donor pool and improving outcomes in an aging population.

## Introduction

A demographic shift is fundamentally altering healthcare systems and the landscape of organ donation and transplantation [1, 2]. Alongside this, the incidence of liver disease continues to rise as a leading cause of morbidity and mortality in the Western world [3–5]. Liver transplantation remains the only curative treatment for patients suffering from end-stage liver disease. Between 2015 and 2050, the proportion of the world’s population aged over 60 years is expected to rise from 12% to 22% [6, 7]. Accordingly, donors over 60 account for more than one-third of all deceased-organ donations in the UK, Eurotransplant area, and the US, further aggravating organ scarcity and waiting list mortality [8–10]. In Europe and North America, the average age of organ donors has increased over recent decades [8–12], and in Scandinavia, it has already reached 61 years [13]. Despite growing demand and organ scarcity, overall organ utilization remains suboptimal, with many older grafts being declined based on chronological age rather than age-related biological metrics [9, 14, 15].

Donor livers from individuals aged ≥65 years are typically categorized as expanded-criteria donors (ECDs). These grafts are associated with an increased risk of early allograft dysfunction (EAD) and inferior long-term outcomes, particularly in the presence of accompanying risk factors such as steatosis, prolonged ischemia, or donation after circulatory death (DCD) [16, 17]. Despite these concerns, ECD grafts represent a substantial yet underutilized source [14, 15, 18, 19] and recent studies highlight that donor age alone should not preclude transplantation [20–23]. Instead, biological aging (manifested through molecular and cellular alterations such as impaired proteostasis, mitochondrial dysfunction, stem cell exhaustion, altered intercellular communication, and senescence) could be considered a more robust metric to inform decision-making in the transplant process.

Although senescence represents only one component of this multifactorial process, causal evidence suggests that targeted elimination or modulation of senescence can delay aging-related decline and improve tissue function, underscoring its relevance for organ regeneration [24–26].

Growing evidence implicates senescence across various stages of the transplant process: from increased burden in aged donor livers and its transfer to recipients, to exacerbation during organ preservation and ischemia-reperfusion injury (IRI) [27–33]. To address these challenges, therapeutic innovations are required. *Ex vivo* machine perfusion (MP) technologies allow dynamic organ preservation while enabling metabolic assessment, and offer a platform for pharmacological interventions towards organ reconditioning [34, 35]. Several landmark studies have demonstrated that MP can recover marginal human livers, enabling successful transplantation [35–40]. In parallel, anti-senescent therapeutics (termed senotherapeutics) such as senolytic and senomorphic agents represent an emerging biologic strategy to target senescence and its deleterious effects [33, 41, 42]. Integrating senotherapeutic interventions into MP platforms offers the potential opportunity to recondition biologically aged, marginal liver grafts, enhance graft resilience, expand the donor pool, and improve transplant outcomes.

## Chronological versus biological aging: redefining transplant criteria

Chronological age does not capture the functional status of tissues or organs. Biological aging, by contrast, reflects the progressive decline in tissue homeostasis, repair capacity, and metabolic function due to accumulating molecular and cellular damage [25, 43]. Although biological aging generally increases with chronological age, its rate and extent are strongly influenced by disease, underlying genetics and environmental stressors [25].

Biological aging encompasses multiple mechanisms including senescence, genomic instability, epigenetic alterations, proteostasis, disabled macroautophagy, deregulated nutrient-sensing, mitochondrial dysfunction, stem cell exhaustion, altered intercellular communication, chronic inflammation, and dysbiosis (thoroughly reviewed in [24, 43, 44]. Together, these processes reflect how cellular and molecular dysfunction accumulate over time driving organ decline.

Within this framework, senescence emerges as a key link between biological aging and functional tissue decline. Senescent cells (SnCs) accumulate with advancing age and pathological stress [45], impair regenerative capacity, and promote chronic inflammation through the SASP. Furthermore, clearance of SnCs delays aging phenotypes and improves tissue function [46–48]. Both lifestyle interventions (e.g., diet, exercise) and targeted interventions (e.g., senolytics, senomorphics) can reduce SnC accumulation and/or attenuate the SASP, indicating the modifiable nature of senescence [49–51].

Organ-specific data indicate that chronological age alone is a poor predictor of organ function. In kidney transplantation, expression of the senescence marker *CDKN2A* (p16^INK4a^) predicts renal allograft dysfunction more accurately than donor age or telomere length [52]. Similarly, telomere attrition has been linked to poor survival in renal transplantation [53, 54]. Aged donor hearts exhibit increased SnC burden and inflammatory injury after transplantation, linking poor graft outcomes to biological aging [55]. In the liver, healthy hepatocytes and cholangiocytes show little age-related telomere shortening, whereas telomere dysfunction and accumulation of SnCs contribute to and increase with fibrosis [33, 56–61].

Collectively, these findings demonstrate that disease burden and tissue-intrinsic damage may drive biological aging beyond chronological aging [62–64]. This is particularly relevant in transplant medicine, where evidence indicates that biological age may better predict organ quality and long-term outcomes than chronological age [24, 65, 66]. Developing appropriate biomarkers for biological age could therefore refine the assessment of donor organ fitness [67].

### Defining senescence

First described by Hayflick and Moorhead in 1961, senescence refers to a state of irreversible cell cycle arrest accompanied by phenotypic changes induced by stressors such as telomere shortening, DNA damage, oncogenic signaling, or oxidative stress [68]. SnCs remain metabolically active and influence the surrounding microenvironment through the secretion of pro-inflammatory cytokines, chemokines, and proteases collectively known as the SASP [69]. At the molecular level, senescence is orchestrated by multiple drivers, with p53/p21 and p16/retinoblastoma (RB) tumor suppressor pathways playing key roles [26, 70].

Senescence plays a critical role in maintaining tissue integrity, as acute senescence facilitates tissue repair and tumor suppression by halting the proliferation of damaged cells and engaging immune surveillance. However, with aging, chronic accumulation of SnCs promotes inflammation, fibrosis, and functional decline [45, 71–73]. Secreted SASP factors can induce paracrine senescence in neighboring cells and impair immune clearance, exacerbating tissue damage and reducing regenerative capacity [74, 75]. According to the threshold model of senescence, once a critical SnC burden is exceeded, SASP propagation together with inadequate immune clearance, drives accelerated biological aging [41, 64]. In transplantation, a young donor with comorbidities, such as advanced fibrosis, metabolic dysfunction (e.g., < high BMI), alcohol use or disorder, or drug-related injury, may already exceed this threshold and display a more pronounced aging-like phenotype than an older but biologically healthier donor. Assessing senescence could therefore help identify the “biological tipping point” between viable and marginal organs, supporting more precise donor selection.

### Assessing liver senescence

Current efforts aim to define precise senescence-associated markers that capture the cellular and molecular alterations most relevant for *ex situ* reconditioning and graft viability [76].

Canonical senescence biomarkers include cell-cycle inhibitors p16^INK4a^ and p21^CIP1^, DNA damage markers (e.g., γH2AX and 53BP1 foci), and components of SASP (e.g., IL-6, IL-8, and MCP-1, among many others). Additional markers include senescence associated-β-galactosidase (SA-β-Gal) activity, telomere attrition, lipofuscin accumulation, increased anti-apoptotic BCL-2 expression and presence of DCR2 in cholangiocytes [45, 77–79].

**FIGURE 1:**
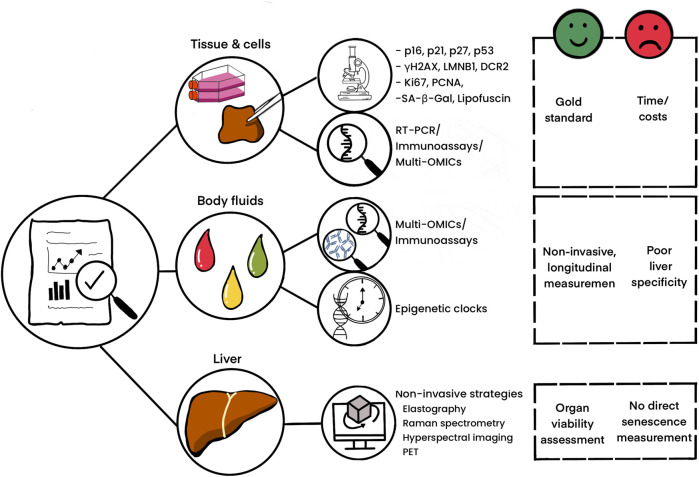
Assessment strategies for liver senescence in the setting of liver transplantation. Assessing senescence in liver grafts during liver transplantation requires complementary approaches. Given the heterogeneity of senescence and the lack of a universal biomarker, multiplex strategies are recommended, in line with the “minimum information for cellular senescence experimentation” (MICSE) framework [70]. In this setting, evaluation may include tissue sampling followed by staining protocols and multi-OMICS analyses, potentially combined with cell culture preparation, as well as the analysis of body fluids (e.g., blood, bile, perfusate, urine) and assessment of graft morphology. Each method offers specific advantages and limitations.

Beyond individual markers, transcriptomic signatures are increasingly applied in experimental and translational transplant settings to capture senescence gene profile across tissues. Single cell sequencing and spatial transcriptomics enable high-resolution mapping of senescent and immune cell heterogeneity in aged or injured liver tissue [26, 33, 70, 78, 80–82]. Moreover, machine-learning-based analysis of histological sections enables automated senescence detection and may have utility in liver graft assessment [83–85] (Figure 1).

Currently no universal senescence marker exists for use in liver grafts and tissue-processing methods affect biomarker reliability [86, 87]. Biomarkers accessible via minimally invasive sampling would be ideal but must account for genetic and phenotypic heterogeneity.

Integrating multi-OMICs profiling and tissue architecture assessment into liver transplant could facilitate the identification of SnC populations amenable to interventions [79].

## Targeting senescence in liver transplant

### Senotherapeutics

The rationale for eliminating SnCs to restore tissue homeostasis emerged from observations that caloric restriction reduces SnC burden and extend health- and lifespan in mice [45]. Furthermore, selective clearance of SnCs delayed age-associated dysfunction in mice [46, 47]. Several approaches have emerged to eliminate SnCs and modulate their effects which can be grouped into the following categories, with Tables 1, 2 summarizing the relevant agents for liver applications.

**TABLE 1:** Selected relevant senolytics and (pre-)clinical findings in the liver.

Agent	Mechanism of action	Pre/Clinical status	Key limitations	Ref.
Dasatinib	Inhibits SRC family kinases → suppressing multiple SCAPs	In combination with quercetin Preclinical: Promotes biliary regeneration in human liver explants and in murine models of cold storage Preservation of cilia morphology and biliary regeneration in human liver grafts Fibrosis suppression and SnCs elimination in murine MASLD model Reduction of hepatic steatosis in murine models Clinical: MASLD (NCT05506488, phase 1 + 2)	Heterogeneous cell-type–specific response; off-target effect and potential systemic toxicity, often combined with quercetin	[29, 30, 51, 88, 89]
Quercetin (natural flavonoid)	Inhibits PI3K/AKT signaling → BCL-2 family → apoptosis	Used in combination with dasatinib to target multiple SCAPs	Low bioavailability; variable potency, combination therapy for efficacy	[28, 29, 50, 90, 91]
ABT-737	BCL-2, BCL-xL, BCL-w Broad BCL-2 family inhibition → induction of mitochondrial apoptosis	Preclinical: Promotes biliary regeneration in murine models of cold storage Improved regenerative capacity following partial hepatectomy in a mouse model Preservation of cilia morphology and biliary regeneration in a mouse model of cholangiocyte senescence	Thrombocytopenia	[29, 32, 51, 91]
ABT-263 (navitoclax- orally bioavailable analog of ABT-737)	BCL-2, BCL-xL, BCL-w Broad BCL-2 family inhibition → induction of mitochondrial apoptosis	Preclinical: Promotes regeneration in acute-on-chronic liver failure in murine models Clinical: Solid tumors (NCT00887757, phase 1) Hepatocellular carcinoma (NCT02143401, phase 1)	Thrombocytopenia Neutropenia	[51, 92–95]
A-1331852	Selective BCL-xL inhibition → induction of mitochondrial apoptosis	Preclinical	Reduced effects of neutrophils - because not targeting BCL-2	[92]
A-1155463	Selective BCL-xL inhibition → induction of mitochondrial apoptosis	Preclinical	Reduced effects of neutrophils - because not targeting BCL-2	[51, 92]
Fisetin (natural compound)	Inhibits PI3K/AKT signaling → BCL-2 → apoptosis	Preclinical:Reduces oxidative stress in liver in senescence mouse models and extends lifespan Reduces inflammation and fibrosis in cholangiocytes senescence mouse model Clinical: Frail elderly syndrome (NCT03430037, NCT03675724) Aging (NCT04994561)	Low bioavailability, variable potency, potential interaction with warfarin; unclear selectivity	[92, 96–98]
FOXO4-DRI peptide	P53 nuclear exclusion in SnCs – cell-intrinsic apoptosis	Preclinical	Currently preclinical	[99]
Procyanidin C1 (natural compound)	Increased ROS production, mitochondria dysfunction	Preclinical: Depletion of SnCs and reduction of SASP and oxidative stress and increase in lifespan in mouse liver	Dose-dependent senomorphic or senolytic effects, high specificity and efficiency, safety	[100]
Piperlongumine	OXR1 degradation, increased ROS generation	Preclinical	Potentially synergistic with ABT-263, currently preclinical, unclear pharmacokinetics	[101]

Abbreviations: MASLD, metabolic dysfunction-associated steatotic liver disease; ROS, reactive oxygen species; SCAPs, senescent cell anti-apoptotic pathways; SnC(s), senescent cell(s).

**TABLE 2:** Selected relevant senomorphics and (pre-)clinical findings in the liver.

Agent	Mechanism of action	Pre/Clinical status	Key limitations	Ref.
Rapamycin everolimus, sirolimus, tacrolimus, RTB101	mTOR inhibitor → NF-κB → SASP suppression	Clinical: Approved for immunosuppression Multiple ongoing studies for aging-related indications	Immunosuppression at higher doses; may require continuous dosing	[102, 103]
Ruxolitinib	Inhibits JAK1/JAK2 → STAT3 signaling → suppress IL-6, IL-8 mediated SASP amplification	Preclinical Clinical: Approved for myelofibrosis, polycythemia vera, GvHD	Broad cytokine suppression; infection risk	[104]
Metformin	Mitochondrial complex I; AMPK → mTOR → NF-κB → SASP suppression	Clinical: Widely used, approved for T2DM. Targeting aging (TAME trial, NCT04245771)	Mild SASP modulation	[105, 106]

Abbreviations: GvHD, graft-versus-host disease; SASP, senescence-associated secretory phenotype; T2DM, type two diabetes mellitus.

Senolytics selectively eliminate SnCs.

SnCs persist within the tissue by activating senescent cell anti-apoptotic pathways (SCAPs), including BCL-2 (BCL-2, BCL-xL, BCL-w), PI3K-AKT signaling, SRC kinase–dependent survival pathways, and cell-cycle checkpoint-associated programs such as CDKN1A-linked signaling [107]. Multi-OMIC analysis has revealed that distinct SCAPs are upregulated across different SnC populations, highlighting the heterogeneity of their survival mechanisms. Senolytics exploit these SCAPs to tip the balance from senescence to apoptosis. Targeting key SCAP nodes using small interfering RNAs induced selective apoptosis in 30%–70% of SnCs while largely sparing non-SnCs [88]. Other senolytics target SnCs through alternative mechanisms, including ferroptosis and metabolic stress responses [90].

Early senolytic discovery followed a hypothesis-driven approach, focusing on natural compounds and repurposed drugs with established safety profiles [41, 88]. More recently, high-throughput screening has expanded the senolytic landscape, enabling the identification of second-generation senolytics with improved selectivity and potency [41, 51, 108].

Senoblockers inhibit upstream senescent drivers, and, unlike senolytics, prevent the formation of new SnCs.

Senoblockers act by interfering with key signaling pathways that induce senescence. These include p53/p21 and p16/Rb axes via selective targeting Hsp72, Bruton’s tyrosine kinase, and the histone demethylase LSD1 among others [109].

Senomorphics (senostatics) do not eliminate SnCs but rather modulate their functional phenotype.

Senomorphics act primarily by suppressing the SASP pro-inflammatory signaling, targeting pathways such as NF-κB, JAK/STAT, mTORC1, p38, and MAPK signaling. Additional senomorphic strategies target mitochondrial complex-I or -IV, modulate NAD^+^/NADH metabolism, or inhibit HSP90 [107, 110]. By exploiting these pathways, senomorphics attenuate the secretion of deleterious cytokines, chemokines, and proteases. By preserving SnCs while limiting inflammatory signaling, senomorphics help to maintain tissue integrity, prevent tumors, reduce senescence footprint and induce regeneration [41].

Immune-mediated clearance of SnCs seeks to enhance the physiological removal of SnCs.

This group includes multiple approaches including immune checkpoint inhibitors, senescence-targeted chimeric antigen receptor (CAR) T cells, vaccines or antibody-drug conjugates, which aim to increase SnC immunosurveillance [111–113].

Certain compounds may exert senomorphic or senolytic effects depending on dose, treatment duration or cellular context. For example, the flavonoid procyanidin C1 is senomorphic at low concentrations and senolytic at higher doses [100]. The translation of these concepts into clinical practice remains in its early stages. To date, human senolytic trial data are limited and predominantly based on Phase I/II studies, demonstrating that intermittent regimens are feasible and well-tolerated. The most studied regimen is oral dasatinib + quercetin (D + Q) for 3 consecutive days repeated in 3-week cycles (D 100 mg/day; Q 1,000–1,250 mg/day), with non-serious adverse events [114, 115]. A landmark Phase II randomized controlled trial demonstrated that response to D + Q is influenced by baseline SnC burden, with significant benefits in individuals with high p16^INK4^ expression [116]. In addition, fisetin has been evaluated at 20 mg/kg/day for 3 consecutive days on an intermittent schedule (NCT04313634).

In liver disease, only one Phase I/II trial of D + Q in metabolic-associated steatotic liver disease (MASLD) has been initiated (NCT05506488) with outstanding results. Looking ahead, the senotherapeutic landscape is expected to expand rapidly.

### Considering risks and limitations

Senotherapeutics offer potential benefits yet entail risks, varying across the different categories:

Senolytics reduce inflammation, tissue dysfunction, and propagation of secondary senescence [41]. This mechanism offers a conceptual advantage over senomorphics, allowing for intermittent, “hit-and-run” administration rather than continuous exposure. However, SnCs can exert beneficial physiological roles (e.g., tumor suppression, promoting wound healing, tissue remodeling and fibrosis resolution) [117, 118]. During wound healing, SNCs can promote closure through PDGF-AA secretion, and senolytic treatment during this period could compromise surgical site healing and anastomotic integrity [119]. Similarly, senescence functions as a tumor suppressor mechanism and prolonged SnC ablation may impair immune surveillance [113, 117, 118]. In the liver, senescence in endothelial cells (LSECs) is protective and compensatory and helps to maintain clearance of toxins during aging [120]. SnC ablation can therefore disrupt tissue homeostasis [32, 121–123]. Many senolytics work within a narrow therapeutic window and are cytotoxic at higher doses [124]. Therapeutic responses may vary according to biological context, sex [121] and senescence heterogeneity [122].

Senomorphics, preserve tissue homeostasis by retaining SnCs while mitigating their harmful effects. However, targeting specific pathways is challenging in a dynamic context where SnCs adaptively alter their SASP in response to the environment. For example, broad suppression of inflammatory mediators may result in off-target effects, including unintended inhibition of cytokine production by non-SnCs, potentially impairing necessary responses [107, 110, 113].

Senoblockers do not eliminate SnCs but prevent new SnC forming, potentially blocking senescent dependent tumor-suppressive functions. Therefore, their efficacy and safety remain to be established [109].

Immune-mediated strategies aim to enhance physiological SnC clearance but face several limitations: immunosenescence and age-related immune dysfunction can reduce the efficiency of SnC recognition and elimination, while the SASP may suppress immune surveillance. CAR-T cell mediated approaches risk off-target immune activation and tissue toxicity [90, 111, 112].

Combination strategies offer promising means to enhance efficacy while limiting toxicity. Combined D + Q treatment targets multiple SCAPs and demonstrates superior efficacy compared with treatment using dasatinib or quercetin alone [88]. Galacto-conjugation and β-galactosidase–activated prodrug strategies have been developed to further improve selectivity [123, 124]. Current evidence relies heavily on rodent data and fails to capture human heterogeneity. Natural compounds such as quercetin and fisetin have variable bioavailability and systemic endpoints are difficult to establish given the wide nature of senescence. The *ex vivo* MP scenario allows a uniquely controlled environment where the graft is isolated and senescence can be measured from biopsies, perfusate, or bile. Drugs delivered directly into the perfusion circuit bypass systemic limitations, allow higher local dosing, and provide a controlled, time-limited treatment window.

## When, what and how: a biomarker-guided, senescence-targeted approach to liver transplantation

**FIGURE 2:**
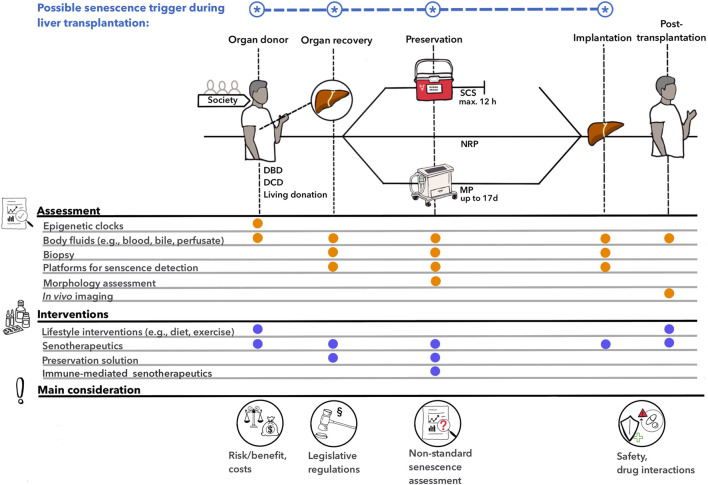
Integrating senescence assessment and targeting along the liver transplantation process. Distinct stages of the transplant process, from donor selection to post-transplant follow-up, provide opportunities for senescence assessment and therapeutic intervention. Senescence is known to be exacerbated at specific points along this pathway, while feasibility of these approaches depends on logistical and ethical considerations. Stage specific considerations are further detailed in Information Boxes 1–5 (Figures 3–7).

There are opportunities to detect and target SnCs during the stages of liver transplantation: donor pool, organ recovery, organ preservation (static cold storage (SCS) and MP), implantation and post-transplantation (Figure 2). Each timepoint offers benefits, logistical and ethical challenges [67, 125].

### Donor pool

**FIGURE 3:**
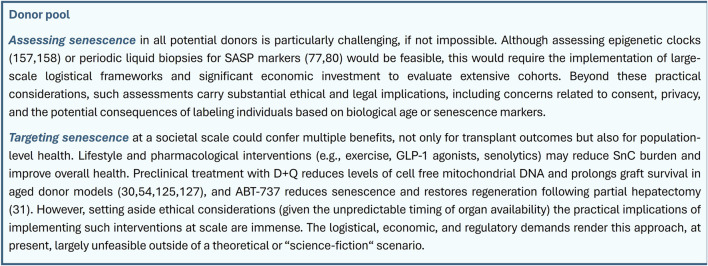
Information Box 1| Assessing and targeting senescence in the donor pool. Conceptual approaches for evaluating and modulating senescence at the population level and in the donor prior to organ procurement.

Strategic considerations for assessing and targeting senescence in the donor pool are summarized in Information Box 1 (Figure 3).

High SnC burden in donors can increase organ immunogenicity, exacerbate alloimmune responses, and ultimately compromise graft function and survival. Importantly, the increased SnCs burden is not limited to the donor organ itself. Emerging data suggest that SnCs can exert systemic, non-cell autonomous effects, propagating the senescent blueprint from graft to recipient, thereby contributing to post-transplant dysfunction. This is exemplified by the transfer of SnCs into young mice, which induces secondary senescence and pathological traits in distant tissues [126–129]. Consistently, cardiac transplantation from aged C57BL/6 donors into young recipients increases SnC burden and SASP across multiple recipient tissues, accompanied by impaired physical performance, and reduced cognitive function within 30 days post-transplantation [31]. Aging also activates profibrotic pathways in the liver, inducing senescence LSECs and increasing SASP, which promotes leukocyte adhesion and chronic inflammation [130]. This highlights the context-dependent role of LSEC senescence: protective for toxin clearance but potentially harmful when excessive or chronic. In addition, hepatocellular senescence induces secondary senescence in other organs, exacerbating systemic inflammation and driving multi-organ dysfunction [131].

### Organ recovery

**FIGURE 4:**
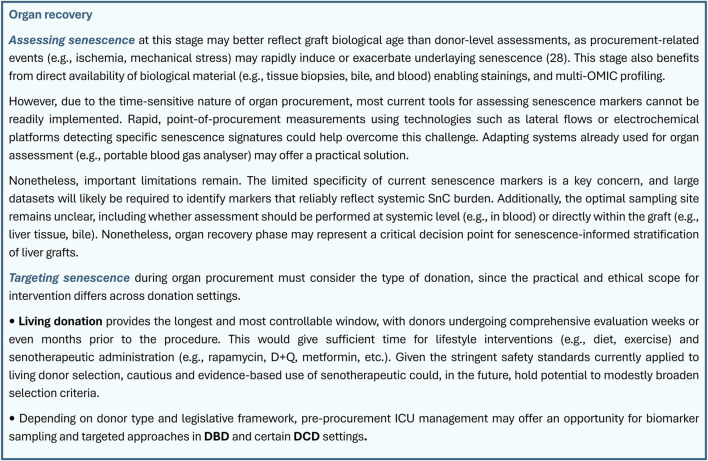
Information Box 2| Assessing and targeting senescence during organ recovery. Potential opportunities for senescence evaluation and intervention at the time of graft procurement.

Potential approaches for senescence assessment and targeting during organ recovery are outlined in Information Box 2 (Figure 4).

Importantly, the senescence landscape at this stage is also shaped by the type of donation. Compared with donations after brain death (DBD), DCD are subjected to warm ischemia prior to cold ischemia, which experimental evidence suggests may increase senescence [29, 30].

Conceptually, several strategies could be integrated into organ recovery, including single or repeated *in situ* graft flushing, delivery of senotherapeutics via regional perfusion circuits, or targeted bench flushing prior to preservation. Pilot studies incorporating quercetin and sucrose [102] or ABT-737 [29] to preservation solutions during SCS and perfusion models showed decrease SnC burden and enhanced regenerative capacity. However, because organs are routinely flushed with preservation solutions and exposed to various medications during standard procurement, concerns remain regarding drug interactions and reduced efficacy under hypothermic, metabolically suppressed conditions.

Alternatively, *in situ* normothermic regional perfusion (NRP), which partially restores circulation and metabolism, could create an additional window for senescence assessment and targeted intervention [132].

### Organ preservation

**FIGURE 5:**
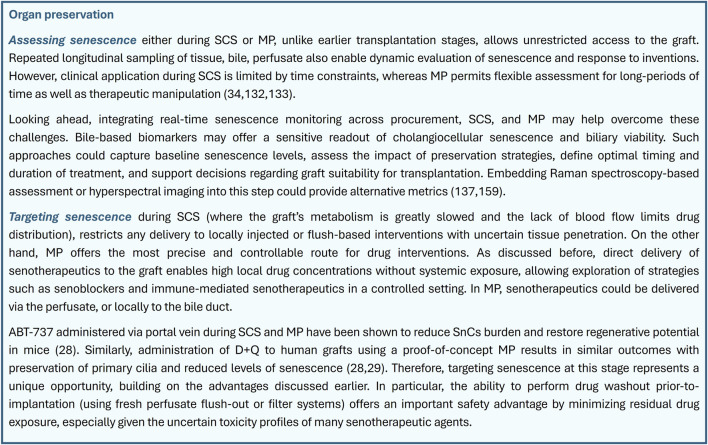
Information Box 3| Assessing and targeting senescence during organ preservation. Experimental and therapeutic opportunities to evaluate and modulate senescence under controlled preservation conditions. This box highlights potential strategies to assess and target senescence during organ preservation.

Following procurement, the graft is fully isolated from both donor and recipient, allowing prolonged organ interventions under highly controlled conditions, offering a unique window for senescence evaluation, organ stratification and targeted therapies. Key opportunities for senescence evaluation and targeted intervention during organ preservation are presented in Information Box 3 (Figure 5).

- SCS elicits distinct, cell-type specific responses within the liver: hepatocytes predominantly undergo apoptosis, while cholangiocytes enter senescence with prolonged cold ischemic times [29]. This has been shown to directly alter/disrupt biliary architecture and impair liver regeneration, potentially affecting post-transplant outcomes. Mechanistically, DCR2 has been shown to play a pivotal role in the development of cholangiocyte senescence [29]. Moreover, prolonged ischemia shortens primary cilia in cholangiocytes (organelles essential for bile flow sensing) and triggers senescence [30]. These findings position organ preservation not merely as a passive holding period, but as an active biological phase during which senescence is induced, amplified, and potentially therapeutically reversible.
- MP offers an opportunity to maintain organs under near-physiological conditions, allowing longitudinal sampling before and after targeted interventions, supporting within-organ comparisons. Emerging approaches, including extended perfusion strategies [35, 133–135] and split-liver techniques [35], also open the possibility of using internal controls to simultaneously evaluate different senotherapeutics. Proof-of-concept studies for *ex vivo* senolytic delivery have shown feasibility in discarded human livers [29, 30] where left lateral segments were perfused with 5 mg/kg dasatinib and 50 mg/kg quercetin. However, systemic application in clinical MP remains in its early stages. Factors such as dosing relative to circuit volume, pharmacokinetics, tissue exposure, timing within the limited perfusion windows are still undefined. Unlike oral trials, perfusion may require single high-dose or repeated bolus strategies to achieve effective tissue concentrations. Perfusion temperature could also strongly influence efficacy. Hypothermia suppresses metabolism, potentially limiting drug uptake and apoptotic signaling, while normothermia preserves cellular activity [136] and could therefore support senolytic actions. Sub-nomorthermic approaches may offer a compromise, but establishing the optimal temperature, timing, and dosing will be essential to advance senotherapeutics in organ perfusion.

### Implantation

**FIGURE 6:**
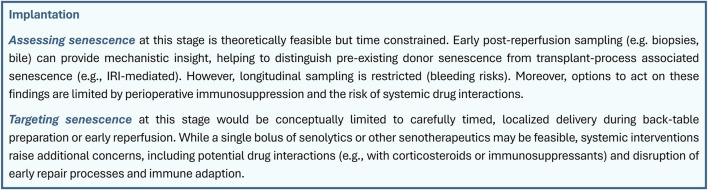
Information Box 4| Assessing and targeting senescence during organ implantation. Considerations for senescence assessment and intervention at the time of graft implantation.

At graft implantation, the preserved organ is revascularized and connected to the recipient’s circulation. Although the graft remains physically accessible, opportunities for interventions are limited: prior to implantation and reperfusion, the organ is ischemic and unlikely to metabolize drugs, and after reperfusion, there is a risk of systemic exposure to any agents administered at this stage. However, IRI is an inherent component of implantation and it has been associated with SnC accumulation and impaired tissue repair, effects that may be reversible with senotherapeutic treatment [28, 137, 138]. Consistently, treatment of rats with rapamycin immediately after kidney transplantation led to reduced senescence and SASP response [139]. Evolving perspectives on senescence assessment and therapeutic targeting at the time of transplantation are detailed in Information Box 4 (Figure 6).

### Post-transplant and follow-up

**FIGURE 7:**
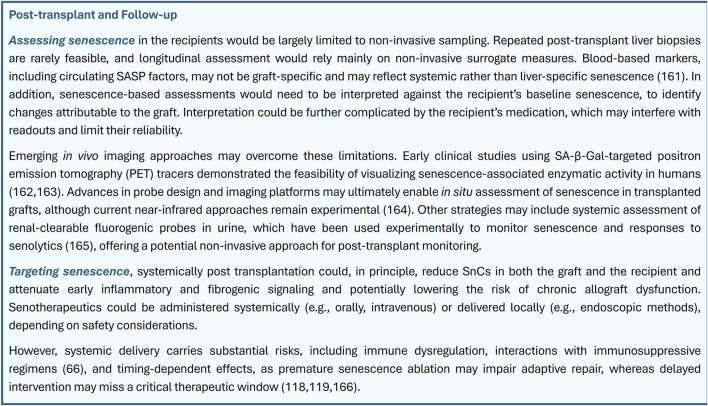
Information Box 5| Assessing and targeting senescence post transplantation. Perspectives on senescence monitoring and modulation after transplantation within the context of immunosuppression.

This stage is crucial for monitoring rejection, optimizing immunosuppression and detecting complications [140]. It represents a phase in which graft-intrinsic biology intersects with recipient systemic factors and determines long-term outcomes. Practical considerations for evaluating and addressing senescence after transplantation are provided in Information Box 5 (Figure 7). SnCs contribute to sustained inflammatory and fibrogenic signaling in graft and recipient. Supporting evidence links senescent cholangiocytes to both acute cellular rejection and chronic rejection in liver transplant recipients and also implicates cholangiocyte senescence as a driver of EAD, long-term graft dysfunction and biliary complications [29, 59, 141–144].

Once maintenance immunosuppression is established, senolytic approaches may be less suitable, and senomorphic strategies may offer a safer alternative. Notably, commonly used immunosuppressive agents, including mTOR inhibitors, exhibit senomorphic properties, suggesting that partial senescence modulation may occur within existing regimes.

Drug-drug interactions remain a key concern [42]. For instance, dasatinib may enhance glucocorticoid-mediated T-cell suppression [145], potentially reducing the need for conventional immunosuppressant but also increasing the risk of over-immunosuppression. Panobinostat may potentiate calcineurin inhibitors (e.g., tacrolimus, cyclosporine) [146], and flavonoids such as quercetin and fisetin may synergize with mTOR-inhibitors (e.g., rapamycin) [147, 148]. While these interactions could theoretically reduce overall drug exposure, they also increase the risk of excessive immunosuppression, opportunistic infection, and malignancy. Conversely, some interactions may be advantageous. For instance, mTOR inhibitors, already used for immunosuppression, possess senomorphic properties, suggesting that partial senescence modulation may be achievable within existing therapeutic regimens without additional agents. Careful dose optimization, immune monitoring, and phase-specific application will be essential.

## Conclusion, challenges, and future directions

Assessing and targeting senescence may represent a paradigm-shifting approach in liver transplantation, potentially improving transplant outcomes and expanding the donor pool.

Adopting a senescence-centered perspective requires re-evaluation of long-standing donor selection criteria.

Chronological age, although widely used as a key selection criterion, may not fully capture organ quality, and alternative metrics (such as biological aging or SnCs burden) may warrant consideration. In this context, donor age can be reframed as a modifiable biological risk factor rather than a fixed contraindication.

Consistent with this view, senescence, a key driver of biological aging, compromises graft quality and propagates dysfunction to the recipient. As such, senescence represents a promising therapeutic target to improve transplant outcomes and expand the donor pool in an aging population [42, 149].

Shifting to biologically informed criteria underscores the need for reliable methods to quantitatively assess senescence in donor organs.

Clinical translation remains hindered by limited understanding of SnC heterogeneity, the lack of specific markers, potential interactions with immunosuppression, and unresolved ethical/regulatory questions. However, multi-OMIC approaches and emerging non-invasive assessment strategies are beginning to address these gaps [81, 150–154]. The field is advancing rapidly, offering great promise, but success will depend on robust safety assessment and the use of biomarkers to guide and monitor interventions. Importantly, distinct liver cell populations (e.g., hepatocytes, cholangiocytes, liver sinusoidal endothelial cells, stellate cells) exhibit different senescent phenotypes with diverse functional impact [130]. Targeting one population may improve certain aspects of organ function while impairing others. Overall, the relative contributions and the consequences of treating these cell types in transplantation remain poorly understood.

Assessing senescence offers a dual benefit: informing organ viability during transplantation and providing a framework to gauge the efficacy of targeted therapeutics. A validated, clinically applicable senescence marker is lacking. Nevertheless, multiple efforts are already underway to target, eliminate, or modulate senescence at different stages of the transplantation process, highlighting the dynamic and rapidly evolving nature of the field.

MP offers a unique platform to address many of these open questions, allowing *ex vivo* evaluation of senotherapeutics without exposing the recipient to systemic effects. Preclinical split-liver models may accelerate development, while early-phase clinical trials with biobanking strategies will be essential to build the evidence base. However, practical challenges must be addressed before routine use. MP itself already represents a substantial cost burden, with internal analyses from a single center reporting an additional ∼10,000 EUR per liver perfusion [155]. Personal demands and institutional demands further compound these challenges. A feasible path forward may involve collaborative multicenter trials conducted at centers with established perfusion programs and research infrastructure already coexist, minimizing duplication of effort and distributing costs across institutions.

Integrating senotherapeutics into MP represents a promising yet evolving strategy to rejuvenate marginal grafts in an aging transplant landscape, with successful translation dependent on rigorous safety assessment and well-designed clinical trials [33, 117, 118].

In conclusion, targeting senescence for organ reconditioning raises the prospect of future organ-repair or rejuvenation centers, where marginal grafts could be biologically optimized before transplantation, requiring collaboration across the transplant community, standardized protocols, and rigorous clinical trials to assess benefits and risks.
